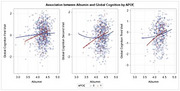# Low Serum Albumin Predicts Accelerated Cognitive Decline in Older Adults with Type 2 Diabetes: Effects of APOE4 Genotype

**DOI:** 10.1002/alz70861_108645

**Published:** 2025-12-23

**Authors:** Hanadi Shamrok, Yuxia Ouyang, Ramit Ravona‐Springer, Tal Davidy, Jack W. Tsao, Yian Gu, Joseph Azuri, Ethel Boccara, Itzik Cooper, Mery Ben‐Meir, Dar Gelblum, Michal S Beeri

**Affiliations:** ^1^ The Joseph Sagol Neuroscience Center, Sheba Medical Center, Ramat‐gan Israel; ^2^ The Icahn School of Medicine at Mount Sinai, New York, NY USA; ^3^ Sheba Medical Center, Tel Hashomer Israel; ^4^ sheba medical center, Ramat gan Israel; ^5^ NYU Grossman school of medicine, New york, NY USA; ^6^ The Gertrude H. Sergievsky Center, College of Physicians and Surgeons, Columbia University, New York, NY USA; ^7^ Maccabi Health Services, Tel‐Aviv Israel; ^8^ The Joseph Sagol Neuroscience Center, Sheba Medical Center, Ramat Gan, Israel Israel; ^9^ The Joseph Sagol Neuroscience Center, Sheba Medical Center, Tel Hashomer Israel; ^10^ Herbert and Jacqueline Krieger Klein Alzheimer’s Research Center at Rutgers Brain Health Institute, New Brunswick, NJ USA

## Abstract

**Background:**

Type 2 diabetes (T2D) is associated with increased risk of cognitive decline and dementia. Low albumin levels may contribute to cognitive decline; older adults with T2D have lower albumin. Low serum albumin is linked to endothelial dysfunction in cerebral blood vessels. This may be a risk factor for neurodegeneration, particularly in APOE4 carriers, who are more susceptible to blood‐brain barrier (BBB) damage.

**Method:**

Participants (*N* =865) were cognitively normal older adults from the Israel Diabetes and Cognitive Decline (IDCD) study. Global cognition and specific domains (episodic memory, executive function, attention, and language) were assessed approximately every 18 months. All participants were clients of Maccabi Health Services, Israel, which provided serum albumin and clinical covariate data since 1998. Linear mixed‐effect models examined associations between baseline serum albumin and cognitive decline. Model 1 adjusted for age, sex, and education; Model 2 additionally adjusted for cholesterol, triglycerides, creatinine, HbA1C, systolic and diastolic blood pressure, and BMI. The interaction between serum albumin and APOE4 genotype was also analyzed

**Result:**

Participants characteristics: mean age 72.27 (SD=4.62) years, 40.6% female, 14.8% ≥ one APOE4 allele, and 13.24 years of education (SD=3.57). Mean HbA1c was 6.83 (SD=0.76). Lower serum albumin was associated with a faster rate of decline in global cognition (Est.=0.0063, *p* <0.0001), executive function (Est.=0.0068, *p* =0.0004), and episodic memory (Est.=0.008, *p* =0.007). No significant associations were found between serum albumin and attention or language. These results remained unchanged after further adjustment in Model 2. A significant interaction was observed between serum albumin and APOE4 genotype, with stronger associations among APOE4 carriers for global cognition (p for interaction=0.0007) and episodic memory (*p* =0.008). No significant albumin × APOE4 interactions were found for executive functions, attention, or language. There were no interactions between albumin and T2D duration or HbA1c. Adjusting for CRP and IL‐6 did not alter the results.

**Conclusion:**

In older adults with T2D, lower serum albumin is linked to faster cognitive decline, particularly in APOE4 carriers. This may result from a compromised BBB in APOE4 carriers, permitting greater albumin leakage into the brain, increasing neurotoxicity and accelerating cognitive decline.